# Comments on the paper “Wolf Shifts and Their Physical Interpretation Under Laboratory Conditions” by K. D. Mielenz

**DOI:** 10.6028/jres.099.024

**Published:** 1994

**Authors:** Emil Wolf

**Affiliations:** Department of Physics and Astronomy, and the Institute of Optics, University of Rochester, Rochester, NY 14627

In a recent paper [1] in the Journal of Research of the National Institute of Standards and Technology, K. D. Mielenz has criticized the generally accepted interpretation of a phenomenon discovered a few years ago, regarding frequency shifts of spectral lines due to coherence properties of sources. In these comments we show that much of the criticism is invalid.

The phenomenon was discovered in 1986 [2] and has been discussed extensively in about 100 papers published since then. In its broader sense the phenomenon concerns the effect of source correlations on the spectrum of the emitted radiation. The paper [1] contains a number of serious errors and misinterpretations, which make much of the criticism invalid or misleading at best.

In the third paragraph of Ref. [1], a passage is quoted from one of my papers on this subject [3] where I suggested some possible mechanisms for producing source correlations. However Mielenz does not state that several such mechanisms have been found since then. Some, in fact, are discussed in several references which Mielenz quotes (his Refs, [8–11]); others are discussed in Refs. [4] and [5] cited below.

Near the beginning of Sec. 2 of Ref [1] Mielenz poses the question “Does the spectrum of partially coherent light change on propagation in free space, and are such changes consistent with the principle of energy conservation?” In spite of Mielenz’s hedging on these questions, the answer to the first question is emphatically “yes, in general,” and the answer to the second question is “yes, always,” That spectral changes can take place on propagation in free-space has been demonstrated most dramatically by experiments of G. Indebetouw [6], in which a partially coherent planar secondary source of essentially uniform spectrum generated light which after propagating in free space exhibited a spectrum which at some points had highly oscillatory behavior. That changes of this kind are consistent with the principle of conservation of energy has been shown in Ref. [7].

In the same paragraph of Ref [1] Mielenz states “The experiments cited here did not pertain to free-space propagation in a literal sense, but were diffraction or interference experiments…”. This statement is simply untrue. Diffraction and interference have played a role in *constructing* the partially coherent secondary sources, but *the propagation from the secondary sources was in free space and has given rise to spectral changes.*

In Sec. 2 Mielenz also states the “spectral distribution…does not change along the path of a ray.” I find it rather astonishing that Mielenz chooses to criticize the interpretation of a rather subtle optical phenomenon by using the most primitive model for light propagation^1^; and what does Mielenz mean by a “ray” in a partially coherent field?

In Sec. 3 Mielenz introduces terms, symbols and notions which follow an International Lighting Vocabulary. Useful as these concepts undoubtedly are for the purposes of practical radiometry, they are largely inappropriate for the analysis of correlation-induced spectral changes in which Interference of partially coherent light, even in free space, demands analysis in terms of statistical wave theory. In this connection it may perhaps not be inappropriate to recall that the foundations of radiometry on the basis of modern theory of radiation has not been fully clarified to this day, in spite of numerous attempts that have been made over a period of several decades^2^. It is also to be noted the term “coherence” is not listed in the International Lighting Vocabulary.

In Sec. 4 Mielenz refers to Newton’s famous experiment on recombination of “colors” dispersed by a prism. Newton’s experiments, classic as they are, are irrelevant to elucidating the effect of correlation-induced spectral changes. The wave theory of light was not even formulated at Newton’s time to explain his observation; and he did not use partially coherent sources.

Near the end of Sec. 4 there appears the statement that “The Helmholtz equations [Eq. (5)] apply to individual frequencies and thus appear to imply that spectra do not change on free-space propagation…”. Evidently appearances can be deceptive, because already in the first publication on this subject (Ref [2]) and in numerous subsequent publications, it was shown that the two Helmholtz equations which govern the propagation of the cross-spectral density do, in fact, predict that, in general, the spectrum of light changes in free-space propagation; and as already mentioned above, such changes have been demonstrated by experiments which have been carried out in several laboratories.

In Sec. 5 Mielenz criticizes an analysis and interpretation given in Refs, [10] and [11] relating to spectral changes that may arise in Young’s interference experiment. He quotes two formulas for the spectrum in the interference pattern [his Eqs. (24) and (27)], which pertain to interference of light from a spatially incoherent source. For this particular case it is true, as Mielenz implies, that the same results can be derived without the use of coherence theory. But he does not quote the more general formulas (Eq. (6) of Ref. [10] and Eq. (3) of Ref. [11]) which cannot be derived in this way. For example, if the light incident on the two pinholes is generated by a laser operating on several modes or has emerged from a rotating ground glass-plate that was illuminated by spatially coherent light, the spectrum of the light in the Young’s interference pattern could not be predicted the way Mielenz suggests but would require the use of the more general formulas.

For essentially the same reasons, Mielenz reference towards the end of Sec. 5 to the well-known textbooks by Jenkins and White and by Strong are inappropriate, as they do not discuss Young’s interference experiments with partially coherent sources.

There is actually much more in Ref. [1] that one might rightly question, but the preceding remarks should suffice to show that Mielenz’s criticism is largely unfounded and unsound, or is confined to special situations involving spatially incoherent sources for which alternative, although equivalent, interpretations of the phenomenon of correlation-induced spectral changes can be given.

I find it regrettable that some members of the radiometry community have adopted a rather negative attitude towards the recent developments relating to radiation from sources of different states of coherence. This is probably due to the fact that they are mainly accustomed to dealing with traditional thermal sources, which are effectively spatially completely incoherent. As already mentioned, the radiation fields produced by such sources can indeed be analyzed by the traditional methods, which do not require the use of coherence theory. However, the radiation properties of many other sources which are used today, such as multimode lasers and various x-ray sources [12–14] can only be fully understood within the framework of the. theory of partial coherence. The discussion of Ref. [1] indicates an attitude which can only inhibit real progress in the development and understanding of such sources and of the radiation which they generate.

## Footnotes

1 As early as 1909 the great physicist H. A. Lorentz stressed on p. 25 in his famous book “The Theory of Electrorns” that “…in general it will not be possible to trace the path of parts of energy in the same sense in which we can follow in their course the ultimate particles of which matter is made up.”

2 Some of the problems encountered in attempts to clarify the foundations of radiometry arc discussed in Ref. [8]. Many of the numerous papers on this subject arc reprinted in an excellent collection edited by A. T. Friberg [9].
