# Supplementary material for: Comments on the paper “Wolf Shifts and Their Physical Interpretation Under Laboratory Conditions” by K. D. Mielenz
Source: J Res Natl Inst Stand Technol. 1994 May-Jun;99(3):281–3. doi: 10.6028/jres.099.024 (PMC8345277; doi:10.6028/jres.099.024)
Supplement: Related texts

# Reply to Professor Wolf’s comments on my paper on Wolf shifts

**Published:** 1994-06-01

**Authors:** Klaus D. Mielenz

**Affiliations:** Alpine Lake Resort, Terra Alta, WV 26764-0402

Since Professor Wolfs comments [1] on my paper on Wolf shifts [2] are rather general in scope, (will attempt to answer them in the order of the issues addressed in my paper.

1. *Does the spectrum of partially coherent light change on propagation in free space?*
  Having reconsidered the paper by Indebetow, my answer remains “no.” Indebetow constructed a source that incorporated prisms and, therefore, changed color when viewed at different angles. But the spectrum depended only on the angle of observation, not on the distance traveled by the light. How can this demonstrate “most dramatically” that “the propagation from the source…has given rise to spectral changes”? A proof that the spectrum does not change may be found in my Eqs. (11) and (12).
  I used the theory of partial coherence, not a “most primitive model,” to derive Eqs. (11) and (12). The word “ray” was a figure of speech, not unlike Wolfs remark that “Newton did not use partially coherent sources.” Does it not follow from the van Cittert-Zernicke theorem that a hole in a window shutter is partially coherent?
2. *Do the theory of partial coherence and the classical Huyghens-Fresnel-Kirchhoff diffraction theory give different results in situations that involve incoherent physical sources? Which of them should be applied for solutions of practical problems?*
  According to my Eq. (21) the theory of partial coherence is a generalization of classical wave optics. “Wolf shifts” produced by apertures illuminated by incoherent sources are equivalent to diffraction phenomena that can be explained classically. Coherence theory is indispensible for the explanation of diffraction phenomena produced by partially coherent sources. As may be seen from the final paragraphs of his “Comments,” Professor Wolf agrees with these answers.
3. *Are traditional radiometric practices afflicted by previously unknown errors due to the partial coherence of light?*
  The “attitude” of radiometrists can be attributed to papers by Professor Wolf and others that were cited in my paper. These papers quoted the results of a doubtful experiment performed in India as evidence that the spectroradiometric scales of national standards laboratories suffer from large “Wolf-shift” errors. The Indian experiment was suspect because it conflicted with classical results quoted in Sec. 5.2 of my paper. This has now been confirmed by Foley and Wang on the basis of coherence theory (see their paper in this issue of the Journal of Research of the National Institute of Standards and Technology).

## References

[1] Wolf Emil (1994). J Res Nail Inst Stand Technol.

[2] Mielenz Klaus D (1993). J Res Natl Inst Stand Technol.
